# Scale-Dependence of Processes Structuring Dung Beetle Metacommunities Using Functional Diversity and Community Deconstruction Approaches

**DOI:** 10.1371/journal.pone.0123030

**Published:** 2015-03-30

**Authors:** Pedro Giovâni da Silva, Malva Isabel Medina Hernández

**Affiliations:** Programa de Pós-Graduação em Ecologia, Departamento de Ecologia e Zoologia, Universidade Federal de Santa Catarina, Florianópolis, Santa Catarina, Brazil; McGill University, CANADA

## Abstract

Community structure is driven by mechanisms linked to environmental, spatial and temporal processes, which have been successfully addressed using metacommunity framework. The relative importance of processes shaping community structure can be identified using several different approaches. Two approaches that are increasingly being used are functional diversity and community deconstruction. Functional diversity is measured using various indices that incorporate distinct community attributes. Community deconstruction is a way to disentangle species responses to ecological processes by grouping species with similar traits. We used these two approaches to determine whether they are improvements over traditional measures (e.g., species composition, abundance, biomass) for identification of the main processes driving dung beetle (Scarabaeinae) community structure in a fragmented mainland-island landscape in southern Brazilian Atlantic Forest. We sampled five sites in each of four large forest areas, two on the mainland and two on the island. Sampling was performed in 2012 and 2013. We collected abundance and biomass data from 100 sampling points distributed over 20 sampling sites. We studied environmental, spatial and temporal effects on dung beetle community across three spatial scales, i.e., between sites, between areas and mainland-island. The γ-diversity based on species abundance was mainly attributed to β-diversity as a consequence of the increase in mean α- and β-diversity between areas. Variation partitioning on abundance, biomass and functional diversity showed scale-dependence of processes structuring dung beetle metacommunities. We identified two major groups of responses among 17 functional groups. In general, environmental filters were important at both local and regional scales. Spatial factors were important at the intermediate scale. Our study supports the notion of scale-dependence of environmental, spatial and temporal processes in the distribution and functional organization of Scarabaeinae beetles. We conclude that functional diversity may be used as a complementary approach to traditional measures, and that community deconstruction allows sufficient disentangling of responses of different trait-based groups.

## Introduction

Community ecology has advanced greatly in recent decades with the understanding that local species diversity is jointly affected by ecological processes operating at different spatial scales [1–3]. This occurs because environmental variables that shape communities differ in their range of variation across spatial scales [4, 5]. The study of the relative importance of ecological processes across different spatial scales in driving local communities is an issue of metacommunity theory [6, 7]. The term ‘metacommunity’ currently refers to a set of communities connected by dispersal of potentially interacting species [6].

Four theoretical models have been proposed to characterize mechanistic processes operating in metacommunities: species sorting, patch dynamics, mass effects and neutral model [2, 6, 7]. These models consider two main issues: whether and how species respond to changes in environmental conditions, and whether species dispersal ability is limited, efficient or high [7, 8]. In heterogeneous environments, differences in local communities caused by environmental filters (e.g., quality and resources) and/or interactions between species characterize a metacommunity guided by species sorting [6]. High dispersal of individuals in heterogeneous environments from source to sink areas may rescue populations in harsh environments (i.e., mass effects) [9]. In a homogeneous environment, competition-colonization trade-offs predict that better competitors should exclude better colonists (i.e., patch dynamics) [6]. In an environment with similar environmental conditions, a neutral metacommunity would be composed of individuals of different species that are similar in their competitive ability, dispersal and fitness; in this case speciation, extinction and dispersal limitation drives variation in local community composition [10]. Mass effects and patch dynamics may be special cases of species sorting, and metacommunities can be neutral or guided by species sorting with limited (patch dynamics, sensu [6]), efficient (species sorting, sensu [6]) and high (mass effects, sensu [6]) dispersal [8]. However, a metacommunity may be structured by more than one paradigm [6], and mechanisms may have greater or lesser importance depending on spatial scale [4]. A key issue is to understand the relative roles of environmental and spatial processes [8].

Temporal turnover may be useful for identifying key processes structuring local communities, although different organisms may respond differently depending on the temporal scale used [11]. This process in species abundance may have a crucial role in ecosystem functioning [12], and needs to be taken into account when assessing environmental effects on biological communities at different spatial scales. Thus, the main goal of metacommunity theory is to explain how the interaction between species dispersal ability and local dynamics influences the structure of biological communities [13].

Over the last few decades, ecologists have developed a variety of ways to measure diversity [14–21] for the purpose of understanding the ecological processes that create and sustain the diversity of biological communities [14]. Spatial and/or temporal variation in the composition and abundance of species between different sites (β diversity) produces a direct link between diversity at the local scale (α diversity) and the species pool at the regional scale (γ diversity) [22, 23]. The importance of spatial processes has become increasingly clear in recent decades due to greater understanding of how environmental heterogeneity and species dispersal ability vary over space, thus promoting differential structuring of local communities depending on scale.

In addition to studies of variation in species composition and abundance, alternative ecological methods have recently been used to investigate community structure. Among them is functional diversity based on species traits [24]. A trait is a measurable variable with the potential to affect the performance and fitness of a species [25]. The trait can be physical, biochemical, behavioral, and phenological or temporal, and in this sense, a species would consist of sets of individuals sharing similar traits [25, 26]. Traits determine when and where species can exist and how they can interact with individuals of other species [26]. Species with similar responses to the environment or similar effects on key ecosystem processes form functional groups [27]. Further, the sets of traits contained within species functional groups may be related to environmental characteristics [28]. Functional diversity is the component of diversity that has the potential to affect the functional dynamics of the ecosystem [29, 30], as well as ecosystem services and processes [31–34]. The functional traits approach also provides a means by which to test the mechanisms driving biological communities, because these mechanisms influence the fitness of the species via the traits they possess [24]. Thus, diversity measures that incorporate species traits may provide novel information on community structure and dynamics and ecological processes beyond what can be determined from the traditional measures generally used in ecology and conservation studies (e.g., composition, abundance and species richness) [26].

Community deconstruction is another method gaining in popularity [35–38], which partitions species-by-site data into subgroups based on species traits. This enables categorizing species into homogenous groups, which can facilitate interpretation of causal mechanisms for species patterns observed in nature [39]. For example, generalist and/or common species generally exhibit broad environmental tolerance while specialist and/or rare species have a specific or narrow tolerance to environmental variation [35, 37]. In general, studies on metacommunities do not distinguish between species and groups of species, even though responses to the environment and population dynamics may be distinct between these organizational levels (e.g., dispersal ability, environmental tolerance) [35]. This approach can also be expanded to other sets of species characteristics that influence life history, such as dispersal mode and body size [38]; this information may provide a better understanding of the relative importance of community structuring processes, particularly for some species groups. Furthermore, the use of these approaches may aid our understanding of scale-dependence of some ecological processes, and may help to determine whether the new methods contribute to our understanding of community structure and the various processes involved.

The objective of this study is to identify the relative importance of environmental, spatial and temporal processes in structuring dung beetle communities at three spatial scales in a mainland-island scenario in Atlantic Forest in southern Brazil, using functional diversity and community deconstruction approaches. As different indices of functional diversity take into account different aspects of communities such as species richness, abundance and evenness [24], we expect that they can serve as a proxy to test the effects of different ecological processes on biological community structure. Deconstructing the entire community using species traits, we expect to find different responses of these groups to different ecological processes [35]. The Atlantic Forest, one of the world’s biodiversity hotspots, is the most endangered Brazilian ecosystem [40], with only roughly 12% of its original size remaining, which is highly fragmented with a high degree of isolation, and with areas mostly in intermediate successional stages [41]. Due to the discontinuous distribution of fragments, the Atlantic Forest offers an interesting model system for the study of ecological processes structuring communities at different spatial scales. Dung beetles (Coleoptera: Scarabaeinae) are excellent model systems for such studies [42, 43], due to ease of sampling with standardized, efficient and inexpensive protocols [44], wide distribution, and high species richness and abundance in tropical regions [45]. These insects respond quickly to anthropogenic environmental changes (e.g., destruction, fragmentation and isolation of forests) with notable changes in species composition, richness, and abundance, and in functional guild proportions [46–50]. Moreover, their diversity is correlated with other taxa, including mammals [48, 51, 52] and are involved in several ecological services such as nutrient cycling, bioturbation (i.e., the displacement and mixing of soil and sediment by animals or plants), secondary seed dispersal and parasite control [53]. Several dung beetle species that inhabit forests avoid distribution into open areas [54–56], and this behavior influences dispersal and colonization when the matrix is inhospitable. Although knowledge of dung beetle dispersal ability is generally scarce, some studies suggest that factors such as sex, body size and perching behavior are related to the movement capacity of these insects [57–59].

We sampled dung beetle communities at 20 sampling sites divided into four large areas of Atlantic Forest, two on the island and two on the mainland in Santa Catarina, southern Brazil, during the summers of 2012 and 2013. The sampling design is hierarchical and the landscape discontinuous, thus it was possible to access the effect of different ecological processes (i.e. environmental filters, spatial structuring and temporal turnover) on dung beetle community structure at three different spatial scales (i.e., sites, areas, mainland-island). We used indices of functional diversity and also deconstructed the community into groups of species with similar traits to test the following hypotheses: (i) dung beetle beta diversity will increase with spatial scale; environmental filters will be most important at a local scale while spatial processes will be most important at larger scales due to the dispersal limitation; (ii) functional diversity will have a similar response to the effects of different ecological processes across spatial scales as do traditionally used metrics (e.g., species composition, abundance, biomass); (iii) the deconstruction of community into groups of species with similar traits will show different responses according to each functional group. We anticipate that trait-dependence will render some functional groups more sensitive to environmental filters (e.g., rare, specialist, diurnal species), and others more sensitive to spatial effects (e.g., common, generalist, nocturnal species) [60]. Overall, these relatively recent approaches will increase the explanatory power of the models and hence, our understanding of the primary mechanisms involved in the structuring of biological communities.

## Materials and Methods

### Study area

**Fig 1:**
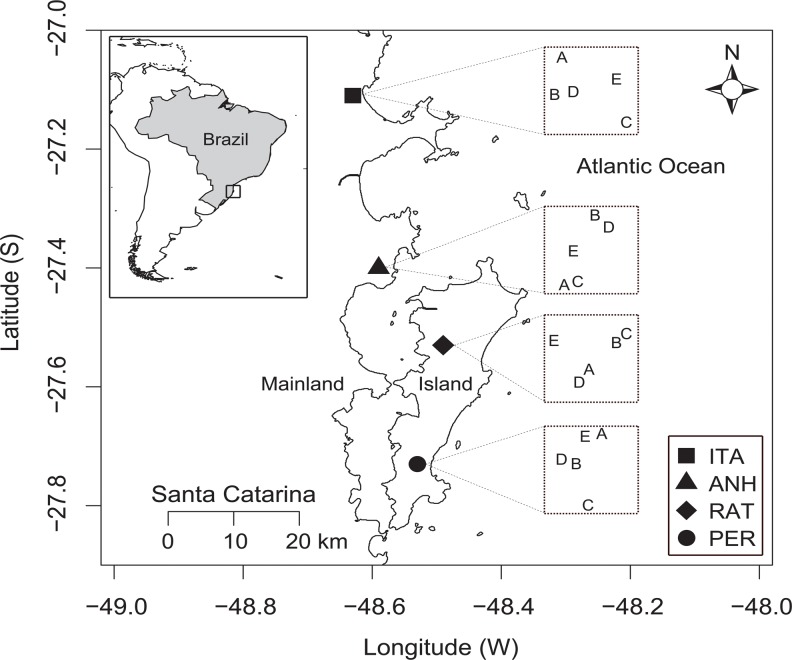
Map of the four areas and schematic distribution of sites sampled (represented by letters A-E, unscaled distribution) in eastern Santa Catarina, southern Brazil. ANH: Environmental Protection Area of Anhatomirim; ITA: Permanent Protection Area of Itapema; PER: Lagoa do Peri Municipal Park; RAT: Permanent Protection Area of Ratones. Reprinted from [5] under a CC BY license, with permission from Pedro G. da Silva and Malva I. M. Hernández, original copyright 2014 (see S3 Fig). Figure is similar but not identical to the original image.

The study sites consisted of four large Atlantic Forest areas in Santa Catarina state, southern Brazil, two on the mainland (both on the east coast) and two on the island of Santa Catarina (municipality of Florianópolis) (Fig 1). The island of Santa Catarina has a total land area of 424.4 km² (54 km north-south, maximum of 18 km wide) and the distance between the mainland and the island varies greatly (minimum 500 m, maximum ~10 km). On the mainland, one study area lies within the Environmental Protection Area of Anhatomirim in Governador Celso Ramos city (ANH, 27º25’1”S, 48º34’25”W), and the other in a Permanent Protection Area in the municipality of Itapema (ITA, 27º05’13”S, 48º35’54”W). On the island, one study areas lies within the Lagoa do Peri Municipal Park (PER, 27°43’30”S, 48°32’18”W) and the other in the Permanent Protection Area of Ratones (RAT, 27°31’52”S, 48°30’45”W). According to the Brazilian Forest Code (Law nº. 12.651/2012), permanent protection areas are sites with characteristics that have the environmental function of preserving water, biodiversity resources, and landscape and geological stability, and for facilitation of floral and faunal gene flow. All sites sampled are near the Brazilian Atlantic coastline, and have dense rain forest vegetation [61] within the Atlantic Forest biome, with various levels of vegetation succession. According to the Köppen classification, the climate in the eastern region of Santa Catarina is Cfa, humid subtropical (mesothermal) with no dry season and hot summers (mean 25°C), and well distributed rainfall throughout the year (app. 1,500 mm annually) [61]. The distance between sites is as follows: PER and RAT, 21 km; PER and ANH, 34 km; PER and ITA, 71 km; ANH and RAT 13.5 km, ITA and RAT, 50 km; ANH and ITA, 37 km. Sampling site altitude ranged between 28 and 265 m.

### Dung beetle sampling

We sampled Scarabaeinae dung beetles using baited pitfall traps made with plastic containers (15 cm diameter x 20 cm depth) buried with the top edge at ground level, allowing beetles to fall in. The traps were protected against rain using a small sheet supported by wooden sticks, placed approximately 10 cm above the trap to prevent overflow. A mixture of water and neutral detergent (300 ml) was added to each container to retain trapped beetles. Human feces and rotting flesh (aged in plastic containers at room temperature three days prior to sampling) were used as bait to attract dung beetles to attract both coprophagous and necrophagous species. Approximately 30 g of each bait type was wrapped in thin cloth and tied in the central part of the rain protection above the traps, preventing the insects from handling the baits. Collected beetles were sorted and dried in an oven (60°C for 72 h), then weighed on a precision balance (0.0001 g). Specimens were identified to species level by expert taxonomists (Dr. Fernando Vaz de Mello, Universidade Federal de Mato Grosso, Cuiabá, Brazil and Dr. David Edmonds, Marfa, Texas, USA) and deposited in the Entomological Collection of the Centro de Ciências Biológicas at the Universidade Federal de Santa Catarina, Brazil. Type specimens were donated to the taxonomic experts for future reference.

The permission to collect dung beetles was issued by Instituto Chico Mendes de Conservação da Biodiversidade (ICMBio/MMA, permit #32333–3 to MIMH) and Fundação do Meio Ambiente (FATMA-SC). The field study did not involve endangered or protected species. S1 Dataset provides the database of values for abundance and biomass of dung beetle species across the study sites.

### Sampling design

Samples were taken at five different forested (hillside) sites within each sampling area. Distance among sites ranged between 300 m to several kilometers within each area. Each site contained five pairs of traps spaced 5–10 m apart, each pair containing both bait types. The pairs of traps were spaced 50 m apart, and were considered one sampling point. The traps remained in the field for 48 h prior to beetle collection. We sampled a total of 100 points in 20 sites distributed among the four areas. The samplings were carried out during the summer of 2012 and 2013 (January and February of both years), because of high temperatures, and it being the period of greatest abundance of dung beetles in southern Brazil [62, 63]. Due to the spatial configuration of our sampling design, the great distance between the four areas, and the effect of spatial discontinuity between the mainland and island, the sampling sites showed a hierarchical distribution. Thus, it was possible to investigate variation in dung beetle communities at three spatial scales (or spatial levels [64]), i.e., mainland-island, between areas, and between sites. A full, detailed description of the sampling design can be found in a previous work [5]. Sites represent the local spatial scale, i.e., the smallest spatial extent in our study that encompasses five sampling points. Areas represent the intermediate spatial scale with five sites per area. Mainland-island represents the regional spatial scale, i.e., the largest spatial extent in our study that encompasses two areas in each one.

### Dung beetle traits

Dung beetle species were characterized in terms of four ecological attributes: food relocation behavior (rollers, tunnelers or dwellers), diet (coprophagous, necrophagous or generalist), activity period (diurnal, nocturnal or diurnal-nocturnal) and biomass (see S1 Table). Protocols for trait assignments are described in S1 Appendix. We also obtained additional information on dung beetle traits from the literature and from consultations with experts, when necessary. These characteristics are widely used to identify the functional groups of Scarabaeinae species and each one has a particular impact on the ecosystem functioning [65].

Food relocation behavior and nesting strategy may alter the relative success of larval and adult dung beetles in modified forests due to abiotic and biotic changes [60]. Roller species form the food source into a ball and roll it on the ground to another location for burial. These species may be affected by differences in the physical structure of the forest floor [60] while dwellers (which nest within the food resource at the site of discovery) are more susceptible to environmental and climate changes. Tunneler species build their nests and bury portions of food in tunnels beneath the resource.

Dung beetles have a broad diet, however most species have evolved to consume mammal feces (coprophagy). Others prefer to eat carrion (necrophagy), and some consume decaying plant matter (saprophagy). Some species are trophic specialists, mainly those that eat fruit or fungi [66, 67]. Due to this variety of dietary preferences, differences in habitat structure may alter food availability in ways that impact dung beetle community structure.

Dung beetle activity is associated with daytime temperatures and humidity, and differences in forest structure may negatively influence the level of activity of diurnal species [68]. Diurnal species often have smaller body size [69, 70] while large-bodied species are often nocturnal [71]. Dung beetle biomass in a given community is mainly derived from nutrients obtained from mammal feces [72]. Individually, biomass can be used as a measure of body size. This trait is positively correlated with dung removal and secondary seed dispersal for large-bodied, nocturnal dung beetles [73, 74], an important ecosystem service provided by these insects. Dung beetle size (and biomass) has been positively correlated with sensitivity to modification [46] and fragmentation [75] of tropical forests. Large-bodied dung beetles show advantages in food acquisition [76], with better competitive outcomes [77] and are also associated with high dispersal rates [78]. We used these sets of traits to calculate four indices of functional diversity (see Functional diversity section).

### Explanatory variables

We measured 20 environmental variables related to habitat structure, to test their influence on dung beetle community structure. Measurements were performed using the adapted point-centered quarter method [79]. Tree, shrub and soil environmental variables were measured in four quadrants as follows: (1) circumference at breast height, (2) height, (3) top diameter, (4) distance away from the nearest tree to the center of cross, (5–8) same measures for trees up to 10 m distance, (9–12) same measures for shrubs, (13) land slope, (14) canopy cover, (15) percentage of leaf litter cover, (16) percentage of green cover, (17) percentage of exposed soil, (18) height of leaf litter, (19) dry biomass of leaf litter, and (20) altitude. The material and methods used to measure these variables are described in S2 Appendix. See also S2 Table for a summary of environmental measures. Differences in environmental conditions (environmental variables measured) among sampling sites is defined as environmental heterogeneity.

We used a method called Principal Coordinates of Neighbour Matrices [80] to create spatial predictors using the *create*.*MEM*.*model* function [4] for the R 3.1.1 program [81], which is suitable for nested sampling designs [80]. This function produces a set of orthogonal spatial variables in a staggered matrix divided by blocks based on the geographical coordinates, number of blocks (or groups of sites) and sampling sites in each block. Each block represents the hierarchical spatial distribution of the sampling points and different blocks receive a value of zero (0) for each spatial variable created. These variables represent spatial relationships among the sampling sites at different scales, and can be used as explanatory variables for community variation [80]. The spatial variables can also represent spatial structures generated by biotic processes, such as dispersal [82]. Dispersal is expected to be high in closest sites and low when sites are more distant [83].

A dummy variable was used to represent different sampling years. Thus, we were able to test and remove the temporal effect from environmental and spatial models when testing their effects using variation partitioning techniques (see Variation partitioning section).

### Data analysis

#### Diversity partitioning

An approach called ‘true diversity’ [17] has been used to partition diversity into its different components in an additive or multiplicative way [18, 84]. We used the additive partitioning approach (γ = α + β1 + β2 + β3) to estimate beta diversity at three spatial scales for the entire dataset, different years and deconstruction approach (see Community deconstruction section). Alpha (α) is the average species richness in local communities, while gamma (γ) refers to the total species richness observed in the entire set of samples. Each component of beta diversity refers to different spatial scales: β1 = between sampling sites, β2 = between areas, β3 = between mainland-island. We used abundance data for the hierarchical analysis of diversity partitioning. We also conducted a separate analysis for functional groups (see Variation partitioning section). These analyses were performed in Partition 3.0 program [85] using an individual-based randomization (N = 999). We used an algorithm to test whether the observed diversity components could have been obtained by a random distribution of individuals between samples at each spatial scale. The statistical significance is obtained by determining the proportion of null values (created by the randomization procedure) that are greater or smaller than the observed values [85].

#### Functional diversity

We used dung beetle traits important for ecosystem functioning to calculate functional diversity, such as food relocation behavior, diet, activity period and dry biomass [60, 65, 86]. We calculated four indices of functional diversity: functional richness (FRic), functional evenness (FEve), functional divergence (FDiv) and functional dispersion (FDis) [87, 88]. FRic is based on the volume of a multidimensional functional space occupied by the species present in a community, and is measured as a convex hull volume [88]. FEve represents the evenness of species abundance distribution in the functional space [88]. FDiv describes how species abundance is spread within the volume of functional trait space occupied by species [88]. FDis is the average distance of the species to the centroid of all species in the multidimensional trait space [87]. Functional diversity analyses were performed with the *dbFD* function using the FD package [89] for R 3.1.1 program [81].

#### Community deconstruction

To assess the effect of the deconstruction of community data we used the variation partitioning procedure (see Variation partitioning section) in different datasets. Based on sets of ecological traits used to calculate the functional diversity, we decomposed the abundance dataset into groups of species. We decomposed the community dataset based on the food relocation behavior (rollers and tunnelers; dwellers are represented by only two species, so we could not use the variation partitioning procedure for this group), diet (coprophages, necrophages and trophic generalists), activity period (diurnal, nocturnal and diurnal-nocturnal), body size (small, medium and large beetles: species with < 10 mg of dry biomass are classified as small, 10–100 mg as medium, and > 100 mg as large [90]).

We also used combinations of food relocation behavior and body size to create new functional groups. Thus, we created four additional groups: large-sized tunnelers, medium-sized tunnelers, large-sized rollers, and medium-sized rollers. Other groups were represented by only one or two species, and thus were not used in the analyses. Combinations of diet and activity period were not used because we expect that these traits are least important for ecosystem functions provided by these beetles. In addition, the majority of dung beetles were attracted to feces (coprophages and trophic generalists) and these resources are both spatially and temporally unpredictable, so the division into trophic categories seems to be less important with respect to ecological functions.

Furthermore, we decomposed the entire metacommunity based on species occurrence to test the prediction that common species are mainly affected by dispersal limitation while rare species are mainly affected by environmental filters [37, 91]. We used the inflection point criterion to define common and rare species [37]. With this approach, we examined a rank abundance curve and used the inflection point of the curve (the region where the curvature changes) to separate common and rare species. We used non-logarithmic abundance values and visually defined the inflection point (see S1 Fig). Thus, species on the left side were classified as common, and those on the right side as rare.

#### Variation partitioning

To test the effect of different sets of predictors on community matrix variation (abundance, biomass, functional diversity, and functional groups) we used a partial redundancy analysis (pRDA) [80] to partition the total variation of response matrices into environmental, spatial and temporal fractions. Despite being criticized [92, 93], variation partitioning has been used in the study of metacommunities for a long time (e.g., [35, 37, 83, 94, 95]). The pRDA allows decomposition of the total variation into fractions that indicate the relative importance of pure environmental predictors, pure spatial predictors, pure temporal predictors, shared portions of variation, and unexplained variation [96]. The analyses of community matrices were performed after Hellinger transformation [97]. We tested for a linear spatial trend and found a significant longitudinal and latitudinal trend for dung beetle abundance data (longitude: F = 22.681, *P* = 0.001; latitude: F = 5.509, *P* = 0.001) and biomass (longitude: F = 5.412, *P* = 0.001; latitude: F = 25.433, *P* = 0.001). We also found a significant longitudinal trend for dung beetle functional diversity (F = 4.040, *P* = 0.015). Thus, all datasets were detrended prior to analyses [80].

For each analysis, a subset of explanatory variables was selected using the forward selection method [98] in order to avoid Type I error and overestimation of the explained variance. This procedure is performed in two steps. First, a model using all explanatory variables is tested, and the analysis continues if the result is significant (*P* < 0.05). After this step, we checked the variance inflation factor (VIF) to identify collinear variables. Variables with higher VIF > 20 were removed [80]. Next, if the result is significant, the selection of variables continues considering the significance level of each explanatory variable, and the adjusted coefficient of multiple determination (*R* ^2^_adj_, or data variation explained by the model) is calculated using all variables (i.e., the full model). If these criteria are not reached, the variables are non-significant and the analysis is terminated. Variable selection was performed separately for spatial and environmental data.

For the functional diversity dataset we conducted a distance-based approach [95] using Euclidean distance, since several functional diversity indices were correlated with species richness. The proportion between the number of species and number of individuals of each functional group can be found in S2 Fig The analyses were performed using R 3.1.1 software [81] and PCNM and packfor packages [99].

## Results

### General results

We sampled a total of 5,794 individuals, belonging to 28 species of Scarabaeinae dung beetles (3,004 individuals and 21 species in 2012; 2,790 individuals and 24 species in 2013, see S3 Table). The largest number of individuals was found on the island (N = 3765). The mainland showed the greatest species richness (S = 22). Among areas, Ratones had the largest number of species (20) and individuals (2,438), while Anhatomirim had the lowest values (S = 13, N = 975). Four species (*Dichotomius sericeus*, *Canthon rutilans cyanescens*, *Canthidium* aff. *trinodosum*, and *Deltochilum morbillosum*) accounted for 77% of total dung beetle abundance. Only seven species were shared between all sample sites. Four species (*Dichotomius sericeus*, *Coprophanaeus saphirinus*, *Canthon rutilans cyanescens*, and *Deltochilum multicolor*) accounted for 83% of the total dung beetle biomass.

### Patterns of beta diversity across spatial scales

**Fig 2:**
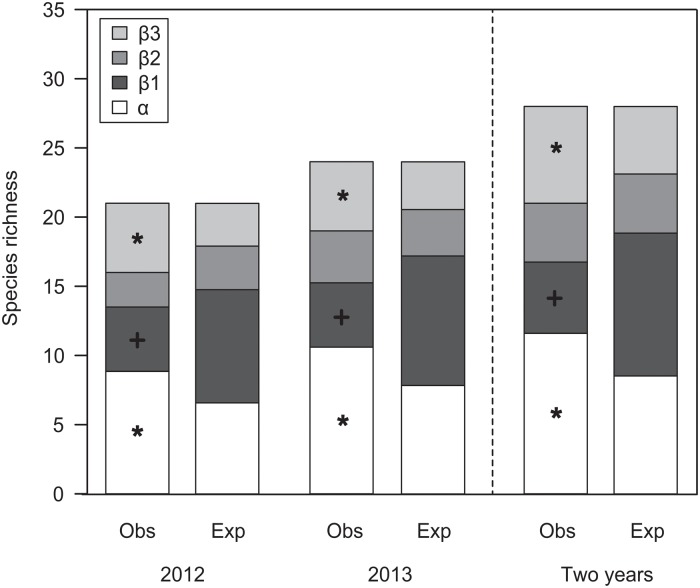
Full hierarchical analysis of diversity partitioning for composition of dung beetles. α = average local diversity, β1 = diversity among sites, β2 = diversity among areas, β3 = diversity among mainland-island. The observed partitions (Obs) are compared with the expected values (Exp) as predicted by the null model based on 999 randomizations. Black star: Exp < Obs, p < 0.05. Black cross: Exp > Obs, p < 0.05.

The total γ-diversity (over two years) was mainly attributed to β-diversity (Fig 2). This was a consequence of the increase in mean α-diversity and β-diversity between areas over the years. The total diversity percentage explained by all β-components was 58.6% (57.8% in 2012 and 55.8% in 2013), of which 18.4% (app. five species) was between sites (β1), 15.2% (app. four species) between areas (β2), and 25.0% (seven species) between the mainland and the island (β3). The total α-diversity was higher than expected by chance (*P* < 0.05) and comprised 41.4% of the total dung beetle species richness, with an average of 12 observed species from the total γ-diversity of 28 species. The α-diversity of pooled 2012 and 2013 data was also higher than expected. The contribution of β-diversity was always higher for β3 and β1 components. Only the observed β-diversity between the mainland and the island was higher than expected by chance. The observed β1-component was nearly always half of the expected. Only β-diversity between areas was equal to the expected value, and always had the lowest contribution to β-diversity among hierarchical levels.

**Fig 3:**
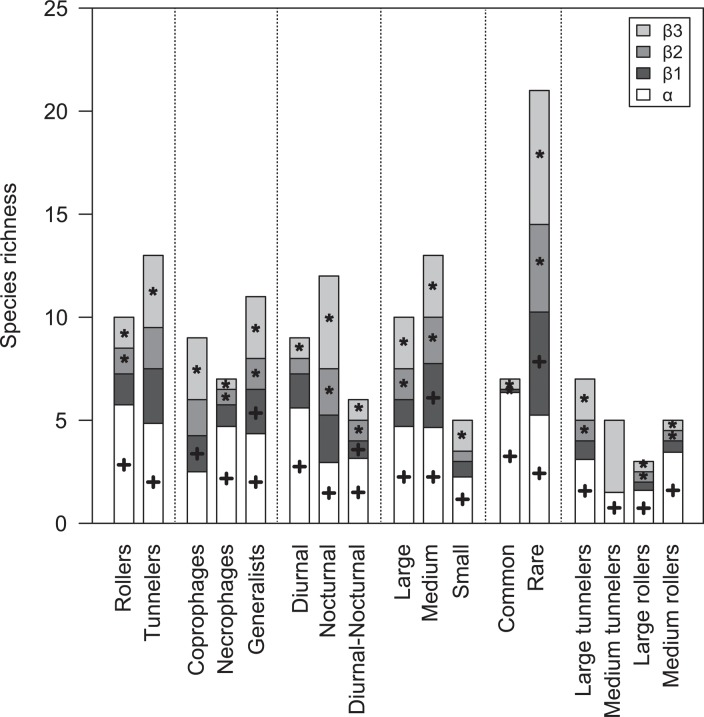
Full hierarchical analysis of diversity partitioning for community deconstruction approach. Diversity partitioning was analyzed for functional groups of food relocation behavior, diet, diel activity, body size, rarity, relocation behavior and size combined. α = average local diversity, β1 = diversity among sites, β2 = diversity among areas, β3 = diversity among mainland-island. The observed partitions (Obs) are compared with the expected values (Exp) as predicted by the null model based on 999 randomizations. Black star: Exp < Obs, p < 0.05. Black cross: Exp > Obs, p < 0.05.

Diversity partitioning of functional groups showed different responses (Fig 3). Out of 17 groups, seven showed greater α-diversity components compared to β-diversity components. The α-component accounted for 90.7% for common species. Medium-sized rollers, necrophages, diurnal species, rollers, large rollers and diurnal-nocturnal species also had high α-diversity. The α-component, however, was always lower than expected by chance.

The highest values of all β-components were found among nocturnal, rare and coprophagous dung beetles. Medium-sized tunnelers, medium-sized, tunnelers, generalists, large-sized tunnelers, large-sized, and small-sized dung beetles also showed higher β-components. In general, the β3-component had the largest values followed by β1-component, with the exception of medium-sized dung beetles. The β3-component accounted for on average 24.2% of the diversity of these functional groups, and was higher than expected by chance for most groups. For medium-sized tunnelers, the β3-component accounted for 70% of the diversity variation. On the other hand, for common species and necrophages it accounted for only 7.1%.

### Environmental, spatial and temporal effects on community variation

#### Traditional vs functional diversity measures

**Fig 4:**
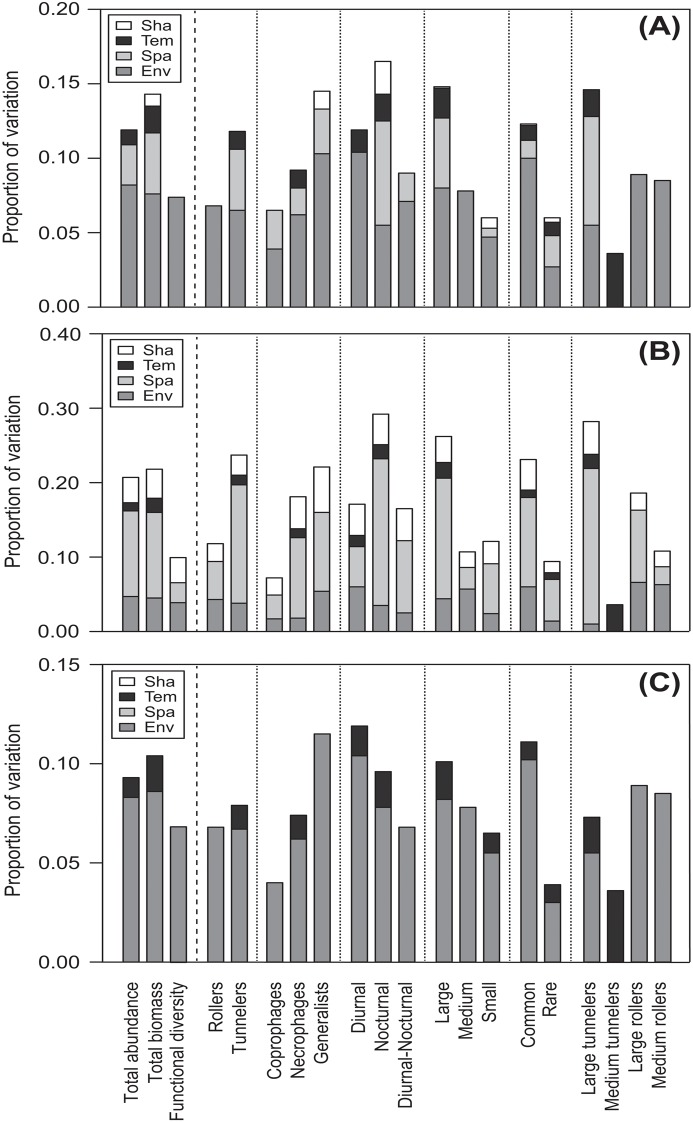
Variation partitioning of the whole dung beetle community (abundance and biomass), the set of functional diversity indices and of communities delimited by their food relocation behavior, diet, activity period, body size, rarity, relocation behavior and size combined across three spatial scales: mainland-island (A), areas (B) and sites (C). Env: pure environmental fraction, Spa: pure spatial fraction, Temp: pure temporal fraction, Sha: shared fraction (all other fractions summed). Right portion after dashed line represents the community deconstruction approach.

Variation partitioning for abundance, biomass and functional diversity showed scale-dependence of processes structuring dung beetle communities using a two-year dataset (Fig 4). At the regional scale (i.e., mainland-island scale) we found a higher and significant environmental effect, followed by spatial and temporal effects that together accounted for 11.9% of abundance variation at this scale (Table 1, Fig 4A). Variation partitioning using biomass data showed the same pattern, but with increased spatial and temporal effects (Table 1, Fig 4A). The explained community variation was also higher, 14.4%. For functional diversity, only environmental effects were important, explaining 7.3% of variation at this scale (Table 1, Fig 4A).

**Table 1:** Results of the partial redundancy analysis for the abundance, biomass and functional diversity of the dung beetle community, and for functional groups composed of food relocation behavior, diet, activity period, body size, rarity, relocation behavior and size combined at the mainland-island scale.

	*P*GEnv	*P*GSpa	*P*GTem	Env Sel^1^	Spa Sel	Tem Sel	E | S + T			S | E + T			T | E + S		
							*R* ^2^_adj_	F	*P*	*R* ^2^_adj_	F	*P*	*R* ^2^_adj_	F	*P*
***Normal approach***															
Abundance	**0.001**	**0.001**	**0.002**	ALT, GC, GTD	1, 2	1	0.082	7.072	**0.001**	0.027	3.954	**0.001**	0.010	3.237	**0.001**
Biomass	**0.001**	**0.001**	**0.001**	ALT, TH, GC, TTD, GTH	1, 2, 3	1	0.076	4.474	**0.001**	0.041	4.067	**0.001**	0.018	5.105	**0.001**
Functional diversity	**0.005**	0.297	0.457	ALT, LS, GC	-	-	0.073	6.017	**0.001**	-	-	-	-	-	-
***Deconstruction approach*** ^2^															
Rollers	**0.001**	0.250	0.512	ALT, GTD	-	-	0.068	8.207	**0.001**	-	-	-	-	-	-
Tunnelers	**0.002**	**0.001**	**0.010**	ALT, TH, LLB	1, 2	1	0.065	5.808	**0.001**	0.041	5.509	**0.001**	0.012	3.661	**0.009**
Coprophages	**0.043**	**0.010**	0.182	ALT	4, 1	-	0.039	9.200	**0.001**	0.026	3.779	**0.006**	-	-	-
Necrophages	**0.001**	**0.045**	**0.016**	ALT	1, 2	1	0.062	14.375	**0.001**	0.018	2.927	**0.004**	0.012	3.538	**0.004**
Generalists	**0.001**	**0.003**	0.136	ALT, GC, TD	1, 3	-	0.103	8.922	**0.001**	0.030	4.411	**0.002**	-	-	-
Diurnal	**0.001**	**0.030**	**0.008**	ALT	-	1	0.104	24.328	**0.001**	-	-	-	0.015	4.345	**0.002**
Nocturnal	**0.001**	**0.001**	**0.006**	ALT, TD, LLB	1, 3, 2	1	0.055	5.315	**0.001**	0.070	6.460	**0.001**	0.018	5.218	**0.001**
Diurnal-Nocturnal	**0.002**	**0.043**	0.394	ALT, GTD, LL	1	-	0.071	6.108	**0.001**	0.019	5.184	**0.003**	-	-	-
Large	**0.001**	**0.001**	**0.002**	ALT, TH, TTD, GC	1, 2	1	0.080	5.575	**0.001**	0.047	6.322	**0.001**	0.020	5.476	**0.001**
Medium	**0.001**	0.213	0.599	ALT, GTD	-	-	0.078	9.447	**0.001**	-	-	-	-	-	-
Small	**0.026**	**0.033**	**0.047**	ALT, GTH	1	-	0.047	5.962	**0.001**	0.006	2.156	0.102	-	-	-
Common	**0.001**	**0.005**	**0.017**	ALT, GC, GTD	1	1	0.100	8.498	**0.001**	0.012	3.662	**0.002**	0.010	3.119	**0.008**
Rare	**0.012**	**0.001**	**0.009**	ALT, GTBA, GTTD	1	1	0.027	2.852	**0.001**	0.021	5.414	**0.001**	0.009	2.821	**0.008**
Large tunnelers	**0.001**	**0.001**	**0.004**	ALT, TH	1, 2	1	0.055	7.251	**0.001**	0.073	9.312	**0.001**	0.018	5.139	**0.004**
Medium tunnelers	0.219	0.969	**0.001**	-	-	1	-	-	-	-	-	-	0.036	8.510	**0.001**
Large rollers	**0.002**	0.090	0.201	ALT, GTBA	-	-	0.089	10.741	**0.001**	-	-	-	-	-	-
Medium rollers	**0.001**	0.236	0.806	ALT, GTD	-	-	0.085	10.236	**0.001**	-	-	-	-	-	-

*P*GEnv: *P*-values of the global environmental models, *P*GSpa: *P*-values of the global spatial models, *P*GTem: *P*-values of the global temporal models, Env Sel: selected environmental variables, Spa Sel: selected spatial variables, Tem Sel: selected dummy variable, *R* ^2^_adj_: data variation explained by the model, E | S + T: pure environmental model, S | E + T: pure spatial model, T | E + S: pure temporal model. *P*-values lower than 0.05 are indicated in bold.

^1^ALT: altitude; GC: green cover; GTBA: greater tree basal area; GTD: greater tree distance; GTH: greater tree height; GTTD: greater tree top diameter; LL: percentage of leaf litter; LLB: leaf litter biomass; LS: land slope; TD: tree distance; TH: tree height; TTD: tree top diameter.

^2^Food relocation behavior: rollers and tunnelers; Diet: coprophages, necrophages and trophic generalists; Diel activity: diurnal, nocturnal and diurnal-nocturnal; Body size: large, medium and small; Rarity: common and rare; Combined functional groups: large tunnelers, medium tunnelers, large rollers and medium rollers.

At the area scale (i.e., intermediate scale), environmental, spatial and temporal models explained significantly variation in abundance (20.7%) and biomass (21.8%) of dung beetles (Table 2, Fig 4B). However, the spatial model was always more important, and the shared fraction also explained a part of total variation for both datasets. At this scale, we found a higher and significant environmental and spatial effect on functional diversity, which alone explained 10% of the data variation (Table 2, Fig 4B). Spatially structured environmental variation also was important for functional diversity at this spatial scale.

**Table 2:** Results of the partial redundancy analysis for the abundance, biomass and functional diversity of the dung beetle community, and for functional groups composed of food relocation behavior, diet, activity period, body size, rarity, relocation behavior and size combined at the area scale.

	*P*GEnv	*P*GSpa	*P*GTem	Env Sel^1^	Spa Sel	Tem Sel	E | S + T			S | E + T			T | E + S		
							*R* ^2^_adj_	F	*P*	*R* ^2^_adj_	F	*P*	*R* ^2^_adj_	F	*P*
***Normal approach***															
Abundance	**0.001**	**0.001**	**0.003**	ALT, GC, GTD	13, 4, 5, 10, 6, 1, 12	-	0.047	4.788	**0.001**	0.115	5.053	**0.001**	0.011	3.599	**0.002**
Biomass	**0.001**	**0.001**	**0.001**	ALT, TH, GC, TTD, GTH	5, 4, 6, 10, 13, 1	-	0.045	3.202	**0.001**	0.115	5.728	**0.001**	0.019	5.622	**0.001**
Functional diversity	**0.001**	**0.003**	0.449	ALT, LS, GC	10, 5, 13	-	0.039	3.698	**0.003**	0.027	2.899	**0.003**	-	-	-
***Deconstruction approach*** ^2^															
Rollers	**0.001**	**0.001**	0.543	ALT, GTD	1, 13, 4, 6, 5, 14, 10	-	0.043	5.708	**0.001**	0.051	2.640	**0.001**	-	-	-
Tunnelers	**0.001**	**0.001**	**0.017**	ALT, TH, LLB	5, 4, 6, 13, 1	1	0.038	4.235	**0.001**	0.159	9.150	**0.001**	0.013	4.231	**0.005**
Coprophages	**0.037**	**0.020**	0.177	ALT	13, 1	-	0.017	4.593	**0.011**	0.032	4.463	**0.003**	-	-	-
Necrophages	**0.001**	**0.001**	**0.014**	ALT	5, 1, 6, 4, 13, 9	1	0.018	5.116	**0.002**	0.108	5.328	**0.001**	0.012	3.927	**0.004**
Generalists	**0.001**	**0.001**	0.132	ALT, GC, TD	13, 10, 4, 6, 12	-	0.054	5.475	**0.001**	0.106	6.348	**0.001**	-	-	-
Diurnal	**0.001**	**0.001**	**0.007**	ALT	13, 5, 1, 4	1	0.060	15.172	**0.001**	0.054	4.234	**0.001**	0.015	4.630	**0.001**
Nocturnal	**0.001**	**0.001**	**0.006**	ALT, TD, LLB	13, 6, 5, 4, 12	1	0.035	4.190	**0.002**	0.197	11.837	**0.001**	0.019	6.151	**0.001**
Diurnal-Nocturnal	**0.001**	**0.001**	0.355	ALT, GTD, LL	1, 6, 13, 5, 4, 9	-	0.025	2.936	**0.001**	0.097	4.809	**0.001**	-	-	-
Large	**0.001**	**0.001**	**0.003**	ALT, TH, TTD, GC	5, 6, 4, 1, 2, 7	1	0.044	3.876	**0.001**	0.162	8.091	**0.001**	0.021	6.329	**0.001**
Medium	**0.001**	**0.011**	0.597	ALT, GTD	13, 14, 10, 6	-	0.057	7.280	**0.001**	0.029	2.623	**0.003**	-	-	-
Small	**0.030**	**0.002**	**0.048**	ALT, GTH	13, 1	-	0.024	3.719	**0.012**	0.067	8.479	**0.001**	-	-	-
Common	**0.001**	**0.001**	**0.013**	ALT, GC, GTD	13, 5, 4, 10, 1, 6, 9	1	0.060	5.935	**0.001**	0.120	5.352	**0.001**	0.010	3.558	**0.004**
Rare	**0.011**	**0.001**	**0.007**	ALT, GTBA, GTTD	4, 13, 6, 1, 5, 3	1	0.014	1.976	**0.009**	0.056	3.027	**0.001**	0.009	2.930	**0.004**
Large tunnelers	**0.001**	**0.001**	**0.006**	ALT, TH	5, 4, 6, 13	1	0.010	2.286	**0.037**	0.209	15.244	**0.001**	0.019	6.114	**0.001**
Medium tunnelers	0.262	0.574	**0.001**	-	-	1	-	-	-	-	-	-	0.036	8.510	**0.001**
Large rollers	**0.004**	**0.001**	0.192	ALT, GTBA	1, 6, 4, 7, 15	-	0.066	8.871	**0.001**	0.097	5.691	**0.001**	-	-	-
Medium rollers	**0.001**	**0.009**	0.790	ALT, GTD	13, 14, 10	-	0.063	7.980	**0.001**	0.024	2.792	**0.005**	-	-	-

*P*GEnv: *P*-values of the global environmental models, *P*GSpa: *P*-values of the global spatial models, *P*GTem: *P*-values of the global temporal models, Env Sel: selected environmental variables, Spa Sel: selected spatial variables, Tem Sel: selected dummy variable, *R* ^2^_adj_: data variation explained by the model, E | S + T: pure environmental model, S | E + T: pure spatial model, T | E + S: pure temporal model. *P*-values lower than 0.05 are indicated in bold.

^1^ALT: altitude; GC: green cover; GTBA: greater tree basal area; GTD: greater tree distance; GTH: greater tree height; GTTD: greater tree top diameter; LL: percentage of leaf litter; LLB: leaf litter biomass; LS: land slope; TD: tree distance; TH: tree height; TTD: tree top diameter.

^2^Food relocation behavior: rollers and tunnelers; Diet: coprophages, necrophages and trophic generalists; Diel activity: diurnal, nocturnal and diurnal-nocturnal; Body size: large, medium and small; Rarity: common and rare; Combined functional groups: large tunnelers, medium tunnelers, large rollers and medium rollers.

At the site scale (i.e., local scale), we found stronger environmental effects on biomass and abundance data (Table 3, Fig 4C). Environmental variables explained 8.3% and 8.6% of abundance and biomass variation, respectively. Temporal effects were also important at this scale, but explained only 1% and 1.8% of abundance and biomass, respectively (Table 3, Fig 4C). Spatial effects were not important for any community dataset. For functional diversity, only the environmental model was important at this scale (Table 3, Fig 4C).

**Table 3:** Results of the partial redundancy analysis for the abundance, biomass and functional diversity of the dung beetle community, and for functional groups composed of food relocation behavior, diet, activity period, body size, rarity, relocation behavior and size combined at the site scale.

	*P*GEnv	*P*GSpa	*P*GTem	Env Sel^1^	Spa Sel	Tem Sel	E | S + T			S | E + T			T | E + S		
							*R* ^2^_adj_	F	*P*	*R* ^2^_adj_	F	*P*	*R* ^2^_adj_	F	*P*
***Normal approach***															
Abundance	**0.001**	0.999	**0.004**	ALT, GC, GTD	-	1	0.083	6.996	**0.001**	-	-	-	0.010	3.141	**0.001**
Biomass	**0.001**	1.000	**0.002**	ALT, TH, GC, TTD, GTH	-	1	0.086	4.793	**0.001**	-	-	-	0.018	4.872	**0.001**
Functional diversity	**0.005**	0.968	0.440	ALT, LS, GC	-	-	0.073	6.017	**0.001**	-	-	-	-	-	-
***Deconstruction approach*** ^2^															
Rollers	**0.001**	0.959	0.546	ALT, GTD	-	-	0.068	8.207	**0.001**	-	-	-	-	-	-
Tunnelers	**0.001**	0.956	**0.015**	ALT, TH, LLB	-	1	0.067	5.763	**0.001**	-	-	-	0.012	3.500	**0.010**
Coprophages	**0.041**	0.160	0.169	ALT	-	-	0.040	9.373	**0.001**	-	-	-	-	-	-
Necrophages	**0.001**	0.985	**0.009**	ALT	-	1	0.062	14.228	**0.001**	-	-	-	0.012	3.470	**0.006**
Generalists	**0.001**	1.000	0.138	ALT, GC, TD	-	-	0.115	9.649	**0.001**	-	-	-	-	-	-
Diurnal	**0.001**	0.541	**0.006**	ALT	-	1	0.104	24.328	**0.001**	-	-	-	0.015	4.345	**0.003**
Nocturnal	**0.001**	1.000	**0.005**	ALT, TD, LLB	-	1	0.078	6.706	**0.001**	-	-	-	0.018	4.814	**0.002**
Diurnal-Nocturnal	**0.003**	0.988	0.381	ALT, GTD, LL	-	-	0.068	5.832	**0.001**	-	-	-	-	-	-
Large	**0.001**	0.999	**0.002**	ALT, TH, TTD, GC	-	1	0.082	5.506	**0.001**	-	-	-	0.019	5.191	**0.002**
Medium	**0.001**	0.922	0.597	ALT, GTD	-	-	0.078	9.447	**0.001**	-	-	-	-	-	-
Small	**0.022**	0.579	**0.048**	ALT, GTH	-	1	0.055	6.805	**0.001**	-	-	-	0.010	3.070	**0.037**
Common	**0.001**	1.000	**0.008**	ALT, GC, GTD	-	1	0.102	8.590	**0.001**	-	-	-	0.009	3.077	**0.007**
Rare	**0.008**	0.978	**0.013**	ALT, GTBA, GTTD	-	1	0.030	3.055	**0.001**	-	-	-	0.009	2.758	**0.004**
Large tunnelers	**0.001**	1.000	**0.005**	ALT, TH	-	1	0.055	6.900	**0.001**	-	-	-	0.018	4.737	**0.004**
Medium tunnelers	0.267	0.431	**0.001**	-	-	1	-	-	-	-	-	-	0.036	8.510	**0.001**
Large rollers	**0.007**	0.774	0.182	ALT, GTBA	-	-	0.089	10.741	**0.001**	-	-	-	-	-	-
Medium rollers	**0.001**	0.946	0.798	ALT, GTD	-	-	0.085	10.236	**0.001**	-	-	-	-	-	-

*P*GEnv: *P*-values of the global environmental models, *P*GSpa: *P*-values of the global spatial models, *P*GTem: *P*-values of the global temporal models, Env Sel: selected environmental variables, Spa Sel: selected spatial variables, Tem Sel: selected dummy variable, *R* ^2^_adj_: data variation explained by the model, E | S + T: pure environmental model, S | E + T: pure spatial model, T | E + S: pure temporal model. *P*-values lower than 0.05 are indicated in bold.

^1^ALT: altitude; GC: green cover; GTBA: greater tree basal area; GTD: greater tree distance; GTH: greater tree height; GTTD: greater tree top diameter; LL: percentage of leaf litter; LLB: leaf litter biomass; LS: land slope; TD: tree distance; TH: tree height; TTD: tree top diameter.

^2^Food relocation behavior: rollers and tunnelers; Diet: coprophages, necrophages and trophic generalists; Diel activity: diurnal, nocturnal and diurnal-nocturnal; Body size: large, medium and small; Rarity: common and rare; Combined functional groups: large tunnelers, medium tunnelers, large rollers and medium rollers.

Altitude, green cover and greater tree distance were the environmental variables selected to compose the environmental model to explain the variation in abundance data, while altitude, tree height, green cover, tree top distance and greater tree height were selected to explain the variation in biomass data. For functional diversity, the environmental variables selected were altitude, land slope and green cover.

#### Deconstructed communities

Variation partitioning of deconstructed communities into species groups with similar traits showed a variety of responses to environmental, spatial and temporal effects (Fig 4). In general, functional groups from a given category (e.g., relocation behavior, activity period, body size) did not show the same response. We were able to identify four response groups (functional groups with similar responses to environmental, spatial and temporal variables) at the regional scale (Table 1). In the first response group, tunnelers and necrophages, as well as nocturnal, large-sized, common, rare, and large-tunneler species were all influenced by environmental, spatial and temporal models. Environmental effects were more important than spatial and temporal effects for most functional groups, with the exception of nocturnal and large tunneler beetles, which were more influenced by spatial effects. The environmental model explained 10% of the variation for common species. The spatial model was more important for large tunneler species, and explained 7.3% of variation. Among these response groups, nocturnal beetles showed the highest total variation explained value (16.5%). The second response group was formed by functional groups that were only influenced by environmental and spatial models. The environmental model was highest for all functional groups. Trophic generalist species showed the highest total explained value of variation (14.5%) and environmental model accounted for 10.3%. Coprophages, diurnal-nocturnal species and small-sized species were part of this response group. The third response group was composed of functional groups that were only influenced by environmental variables. Rollers, medium-sized species, large-sized rollers and medium-sized rollers were part of this response group. Among these, large rollers showed the highest explained value of variation (8.9%). The fourth response group was formed by remaining functional groups that showed differential responses to explanatory models. Diurnal beetles were influenced by environmental (10.4% of variation) and temporal (1.5% of variation) factors, while medium-sized tunnelers were influenced only by temporal factors (3.6%).

At the intermediate scale, we found a higher spatial effect for most functional groups (Table 2, Fig 4). Only diurnal, medium-sized, and medium rollers showed a higher environmental effect. We could find three distinct response groups at this scale. The first response group is formed by functional groups where environmental, spatial and temporal effects were important. Tunnelers, necrophages, diurnal, nocturnal, large-sized, common, rare and large tunneler species were part of this response group. Among these, the spatial model explained 20.9% of large tunneler variation. The greatest amount of variation explained among all models was found for nocturnal dung beetles with 29.2% of the total variation. The second response group was formed by rollers, coprophages, trophic generalists, diurnal-nocturnal species, small, medium and large sized species, and medium-sized rollers, which were influenced only by environmental and spatial models. Only medium-sized and medium-sized roller species showed a higher explained value of variation by environmental models. The third response group was composed of medium-sized tunnelers, which showed different responses. Tunneler species were influenced only by temporal factors (3.6%). At this scale, the shared fraction was very important for most functional groups, showing a large amount of spatially structured environmental variation within the four areas sampled.

At the local scale, only the environmental and temporal models were important for the variation in community data (Table 3, Fig 4). We could identify two main response groups at this scale: those that are influenced only by environmental variables, and those influenced by environmental and temporal variables. Rollers, coprophages, trophic generalists, diurnal-nocturnal species, medium-sized species, and large and medium rollers were influenced only by environmental variables. Among these, the highest explained value was found for generalist species, where the environmental model accounted for 11.5% of the variation in the data. Both environmental and temporal factors influenced tunnelers and necrophages, as well as diurnal, nocturnal, large-sized, small-sized, common, rare, and large tunneler species. The temporal models always had lower values than the environmental models. Among these response groups, diurnal beetles had the greatest explained value and the environmental model accounted for 10.4% of variation in the data set. The shared fraction was not important at this scale, showing negative values.

Taking into account the responses of functional groups across the three spatial scales studied, we identified the occurrence of two major groups of responses (Table 4). The occurrence of significant temporal effect at any spatial scale was used to separate the two major response groups. Each response group was divided into two subgroups according to the variation in the relative importance of environmental, spatial and temporal models, and a brief summary of the relative importance of explanatory models according to each functional group is provided (Table 4). We found few shared dung beetle species for most functional groups (see S1 Table), which demonstrates support for independence of group responses to environmental, spatial and temporal effects.

**Table 4:** Summary of the relative importance of explanatory models according to the different community datasets, and according to each functional group across the spatial scales studied. Groups were formed by similar responses.

**Datasets**	**Increasing spatial scale**			**Group** ^1^ **/Subgroup**	
	**Sites**	**Areas**	**Mainland-Island**		
**Abundance**	Env + Tem	Spa + Env + Tem	Env + Spa + Tem	-	-
**Biomass**	Env + Tem	Spa + Env + Tem	Env + Spa + Tem	-	-
**Functional diversity**	Env	Env + Spa	Env	-	-
**Common**	Env + Tem	Spa + Env + Tem	Env + Spa + Tem	G2	SG1
**Coprophages**	Env	Spa + Env	Env + Spa	G1	SG2
**Diurnal**	Env + Tem	Env + Spa + Tem	Env + Tem	-	-
**Diurnal-Nocturnal**	Env	Spa + Env	Env + Spa	G1	SG2
**Generalists**	Env	Spa + Env	Env + Spa	G1	SG2
**Large**	Env + Tem	Spa + Env + Tem	Env + Spa + Tem	G2	SG1
**Large rollers**	Env	Spa + Env	Env	G1	SG1
**Large tunnelers**	Env + Tem	Spa + Env + Tem	Spa + Env + Tem	G2	SG2
**Medium**	Env	Env + Spa	Env	G1	SG1
**Medium rollers**	Env	Env + Spa	Env	G1	SG1
**Medium tunnelers**	Tem	Tem	Tem	-	-
**Necrophages**	Env + Tem	Spa + Env + Tem	Env + Spa + Tem	G2	SG1
**Nocturnal**	Env + Tem	Spa + Env + Tem	Spa + Env + Tem	G2	SG2
**Rare**	Env + Tem	Spa + Env + Tem	Env + Spa + Tem	G2	SG1
**Rollers**	Env	Spa + Env	Env	G1	SG1
**Small**	Env + Tem	Spa + Env	Env + Spa	-	-
**Tunnelers**	Env + Tem	Spa + Env + Tem	Env + Spa + Tem	G2	SG1

Env: pure environmental model, Spa: pure spatial model, Tem: pure temporal model, G: group, SG: subgroup.

^1^G1: response group more influenced by environmental than spatial processes, and not influenced by temporal processes; SG1 (G1): subgroup where spatial effects were important only at the intermediate scale; SG2 (G1): subgroup where spatial effects were important at the intermediate and regional scales, being spatial effects more important than environmental ones at the intermediate scale; G2: response group also influenced by environmental, spatial and temporal processes; SG1 (G2): subgroup where spatial effects were more important than environmental and temporal ones at the intermediate scale, and environmental effects were more important than other at the regional scale; SG2 (G2): subgroup where spatial effects were more important than environmental and temporal ones at the intermediate and regional scales.

The variables that comprised the environmental models differed among response groups. However, altitude was included as a variable in all models. Greater tree basal area, greater tree distance, greater tree height, green cover, leaf litter biomass, percentage of leaf litter, tree distance, tree height, and tree top diameter were the variables that comprised the environmental models, yet they did not show any pattern among the aforementioned groups.

Comparing the responses of different community datasets (functional groups) with abundance response, we found that data on nocturnal, large-sized, large-tunnelers, trophic generalist, and common species, as well as biomass, showed higher explained values of variation than did abundance at the regional scale (Fig 4). At the intermediate scale, nocturnal, large-sized tunneler, large-sized, tunneler, common, and trophic generalist species and biomass had higher overall explained value of variation than did abundance alone (Fig 4). At the local scale, the functional groups that had higher explained values than abundance were trophic generalists, diurnal, nocturnal, common, and large-sized species, and biomass (Fig 4). In general, data on nocturnal species, trophic generalists, large-sized and common species, and biomass showed higher explained values of variation than did abundance at all three spatial scales studied. Large tunnelers also had the highest values at regional and intermediate spatial scales.

## Discussion

Our results show that environmental, spatial and temporal processes play different roles in structuring species composition in Scarabaeinae metacommunities. However, the relative importance of these processes depends on spatial scale and the community dataset (or species groups) analyzed. Several ecological processes are scale-dependent, showing spatial and temporal differences from local to continental scales [100, 101], and there is a large body of evidence that supports this claim for several groups of organisms in different ecosystems (e.g., [4, 5, 83, 102, 103]). Besides improving our knowledge of scale-dependence of ecological processes in Scarabaeinae metacommunities, our study was able to identify similar responses of functional groups with different species composition and sets of traits. Our results also show that functional diversity metrics are appropriate for the investigation of different ecological processes over increasing spatial scales.

Abundance and biomass data were influenced in the same way by different sets of predictors across spatial scales. At the local scale, environmental and temporal predictors were important. Spatial factors were most important at the intermediate scale, i.e. within areas. Contrary to our expectations and consistent with some other studies, there was a greater environmental than spatial effect at larger spatial scales [4]. These results demonstrate that some environmental variables may show a large spatial variation that can affect species distribution both locally and regionally. At intermediate spatial scales, environmental filters were less important, and spatial processes other than dispersal limitation were more important in structuring dung beetle communities.

Dung beetle biomass is mainly derived from nutrients obtained from mammal feces [72]. Biomass can be used as a measure of beetle body size, which is a trait positively correlated with the ecological functions of dung removal and secondary seed dispersal for large-bodied, nocturnal dung beetles [73, 74]. Our results show that abundance and biomass data respond similarly to the ecological gradient, but that biomass showed a higher value explained by sets of explanatory variables than abundance data. The environmental model tested against biomass data had five significant environmental variables, two more than the environmental model tested against abundance data. So, biomass data may be used as a representative measure of species responses when one is trying to describe environmental and spatial effects on ecological functions of dung beetles.

Functional traits and functional diversity measures are increasing among community ecology studies [24]. These approaches have been applied to different biological groups to investigate the relationships between biodiversity and ecosystem processes [29]. Our results showed that a distance-based functional diversity approach responds differently to environmental, spatial and temporal processes compared to traditional measures such as species abundance and biomass. The environmental model was more important than the spatial model, and there was no temporal effect in functional diversity. Environmental effects may be intuitively more important for functional structure than taxonomic structure (see also [104]), and contrary to old ideas (see [105]), functional structure may be spatially structured. At the intermediate spatial scale, the shared fraction was also important, as in other studies [95]. The absence of a temporal effect can be interpreted as a non-significant temporal turnover of functional diversity, which can be explained by the slight increase in β-diversity between years at all spatial scales. As we demonstrated, general patterns of functional diversity can be influenced by environmental and spatial factors [95, 106, 107] that are dependent on spatial scales. Investigation of the importance of environmental and spatial processes in explaining functional diversity across spatial scales is a recent approach [95, 108]. In our study, we did not investigate the response of each functional diversity index because we were attempting to test the use of a set of indices that take into account different features of communities to be used as proxy for traditional measures. We know that different individual functional diversity indices may respond differently to environmental and spatial predictors, and that they may be scale-dependent [39, 95, 106, 109]. We argue that functional diversity is a complementary tool to answer ecological questions [24, 110] regarding species distribution in the metacommunity framework.

Based on our community deconstruction approach, we were able to identify two main robust response groups, each with two subgroups according to their responses to explanatory models at each spatial scale. The two main response groups are formed by functional groups that were influenced only by environmental and spatial patterns at intermediate scales (group 1) and by the three sets of explanatory predictors at higher spatial scales (group 2). Group 1 can be divided into functional groups that showed a higher importance of spatial effects at the intermediate scale (subgroup 1), and those that also showed greater importance of environmental than of spatial effects (subgroup 2). Subgroup 1 was composed mainly of roller groups and medium-sized dung beetles (composed of rollers, tunnelers and dwellers). Subgroup 2 was formed by coprophages, trophic generalists and diurnal-nocturnal dung beetles, and by unrelated groups. Group 2 showed greater environmental than temporal effects at the local scale. It also showed a greater spatial, followed by environmental and temporal effects at the intermediate scale. At the mainland-island scale the environmental effects were higher than spatial and temporal ones. Moreover, the subgroups can be identified by their different responses at the regional scale; subgroup 1 showed a higher environmental effect while subgroup 2 showed a higher spatial effect.

Coprophages and trophic generalists showed higher β-diversity components than necrophages, and in general, the first groups were represented by more species than necrophages. This is a common pattern found in Scarabaeinae communities in Neotropical and Southern Asia regions [111, 112]. Among these groups, trophic generalists showed the highest value of variation explained by environmental and spatial filters. Necrophages differed from the other two groups because they showed a significant temporal turnover at all spatial scales, despite the importance of spatial and environmental effects at higher scales. The temporal turnover in necrophages may be associated with increased abundance of *Canthon luctuosus* and *Coprophanaeus dardanus*, and with decreased abundance of *Coprophanaeus saphirinus* and *Deltochilum rubripenne* at the same sites and at all spatial scales. Mammal feces and carrion were expected to be spatially and temporally unpredictable. However, we expect that dung resources occur more frequently and is more abundant than carrion. Moreover, carrion is also consumed by other organisms such as large birds and also mammals, whereas dung is utilized almost exclusively by a few insect groups, many of them predators of other insects. So, necrophagous beetles may also be responding to temporal effects such as low availability of food resource, which is well recognized to be one of the most important drivers of dung beetle communities (beyond changes in vegetation structure) [42, 46].

Groups based on activity period showed different responses. Activity period of Scarabaeinae beetles is associated with daytime temperatures and humidity, and differences in forest structure may negatively influence the activity of diurnal species [68]. Diurnal species often have smaller body size [69], while large-bodied species are often nocturnal [71]. Diurnal activity may be a limiting factor for species dispersal when climatic conditions are unfavorable. For example, very warm temperatures, low humidity and strong winds can influence the flight capacity of beetles, even within forests [113]. However, our results showed that nocturnal species were more influenced by spatial filters than were diurnal species, mainly at larger spatial scales. Diurnal species were more influenced by environmental than spatial filters. Diurnal-nocturnal species showed an intermediate response between diurnal and nocturnal species. Diurnal beetles showed a high proportion of species richness due to α-diversity (62.2%), while diurnal-nocturnal species showed similar values of α- and β-components. β-diversity components accounted for 75.4% of species richness of nocturnal beetles, which may explain the higher spatial effect on this group, mainly between areas. We expect that species with different sets of ecological traits have different dispersal abilities, and thus they are influenced by environmental and spatial filters differently [5].

Body size in dung beetles is an important trait that can be affected by modification [46], fragmentation [75], and isolation [114] of tropical forests. Large-bodied dung beetles perform better in dung removal and secondary seed dispersal than do small-sized dung species [73, 74] because they are better competitors [76, 77]. Large-sized dung beetles are also expected to be good dispersers [78]. Our results showed that these beetles were very influenced by spatial factors at higher spatial scales, demonstrating dispersal limitation or other spatial mechanisms that limited their spatial distribution. Environmental effects were important at regional scales, and can play an important role in the distribution of these beetles. Large, medium and small-sized dung beetles also showed similar α- and β-component proportions.

Rollers were influenced mostly by environmental filters. Tunnelers were very influenced by spatial factors at the intermediate scale. Rollers showed a higher α-component while tunnelers showed a higher β-component. Using body size and food relocation behavior combined, we found that large tunnelers and medium tunnelers showed higher β-components. However, their responses to environmental, spatial and temporal processes were very distinct. The spatial effect becomes very important for large tunnelers when these traits are combined. Medium-sized tunnelers were only influenced by temporal effects, and only the β3-component was important. This result demonstrates a temporal turnover at the regional scale for this group; environmental and spatial effects were not important. Large rollers and medium rollers showed higher α-component. Spatial effects were important only at the intermediate spatial scale, and mostly for large rollers. Food relocation behavior alone showed no differences in the responses between rollers and tunnelers, however when combined with body size we found different responses between species with distinct sets of traits. The functional group assignment using sets of traits seems to be a more realistic approach for use in community deconstruction. However, this approach may only be feasible when there are a limited number of traits. If we used all measured traits, we would have 22 different groups from 28 species sampled, and most of them would be formed by one to three species. This would preclude the implementation of multivariate analyses and hamper the gathering of species response patterns. The diversity of biological traits originated by ecological, evolutionary and historical processes is one of the characteristics that are associated with the evolutionary success and high diversity of dung beetles [111]. We expect that the high diversity of traits that can be used in studies like ours is shared by the great majority of organisms, and that this approach may be particularly appropriate for groups with higher species richness.

Common and rare dung beetle species showed the same responses to ecological processes across the three spatial scales. However, the explanatory value was much higher for common species at all spatial scales. Our results also showed that common species have a very low β-diversity, while the composition of rare species is strongly dominated by β-diversity in all its components. Assumptions of classical ecological theory and metacommunity framework suggest that common and rare species should respond differently to environmental filters and dispersal limitation [37, 91]. Furthermore, common and rare species are expected to differ in functional traits and environmental preferences. However, our study (see also [37, 91, 115]) showed similar responses across environmental and spatial gradients in common and rare species. These results may have several explanations. First, rare species are expected to exhibit a higher level of environmental specialization, and can be more affected by spatially structured environmental filters than are common species. Second, common and rare species may respond similarly to environmental factors, but in different ways. For example, for both common and rare species the environmental model was formed by three variables, but altitude was the only one shared by both models. Thus, common and rare species may be affected by different environmental filters that are spatially structured in the same way. On the other hand, rare species may be affected by environmental variables that are difficult to measure [37] and are thus ‘hiding’ the real effect of environmental factors on the group.

Another important factor to be considered is undersampling of species. Species that are considered rare may simply be undersampled due to the inefficient methods. Among dung beetles, many species considered trophic specialists of resources different than those used as bait are typically undersampled, even using standardized and suitable methods. Species rarity is a difficult concept [116] and understanding the mechanisms driving the distribution of rare species is still a challenge in community ecology. Large-scale diversity patterns in aquatic metacommunities can only be well-described using information from common species [115]. Our results indicate the same, but removing rare species does not improve the outcome of analyses when comparing the responses of abundance between rare and common datasets. Species rarity is important in the context of conservation [117] and must be considered when the objective of the study involves the maintenance of biological diversity along ecological gradients, especially anthropogenically altered environmental gradients. However, ecologists should keep in mind that species rarity in disturbed habitats may generate an overestimation of the conservation value of these environments, because the presence of rare species may simply be a sampling artifact [117].

Among the general patterns, we found that environmental effects are prevalent at the local scale, which demonstrates the power of species sorting in local structuring of communities [118]. We also found strong environmental effects on many groups at the regional scale. There is a great body of evidence showing the predominance of environmental filters among aquatic and terrestrial metacommunities [37, 38, 94]. The importance of spatial effects did not follow the increase in spatial scale, and spatial effects were very important at the intermediate spatial scale. This demonstrates that even in the same large forest fragment, the dung beetles “suffer” with dispersal limitation. However, dispersal limitation, if it exists, should be visible at the largest spatial scale studied [4, 102]. Another possibility is that sites close to each other exchange large numbers of individuals and, hence, show mass effects at the intermediate spatial scale (see [83]). We do not have enough data on dispersal of dung beetle species to distinguish between dispersal limitation and mass effect, although the former is more likely due to the greater environmental effect at the local scale and large distance between sites. Thus, Scarabaeinae beetles show a spatially structured community possibly due to the large variation in environmental variables of the sites sampled. These effects are also important at the regional scale, as well as dispersal limitation (or other spatial effect) at intermediate spatial scales, culminating in the greater β-diversity found between the mainland and the island. The temporal effect was also important for the dung beetle community structure, as demonstrated for other groups [119, 120].

The high proportion of the residual fraction is common among metacommunity studies using variation-partitioning methods. A probable cause is that the communities are generally composed of many rare species, which have distributions that are difficult to model [37]. Moreover, snap-shot sampling surveys may yield weak patterns, which are not perfectly structured and may vary in time [110]. Another probable cause is the lack of key explanatory variables, which can be difficult to measure (e.g., biotic interactions) [37]. We measured 20 environmental variables that we expect describe properly the forest structure and environment of the sampling sites. Among the 12 that were selected to compose the different environmental models, the most important variables were altitude, greater tree basal area and green cover. Some of these variables were also related to the distribution of dung beetles in Atlantic Forest fragments in southern Brazil [62, 90]. Altitudinal variation is a common feature in the Atlantic Forest, which is generally composed of mountain chains with different elevations. This feature of the landscape can influence other characteristics of the forest differently, since the land slope was also important for the distribution of functional diversity in our study.

In summary, our study increases evidence of the importance of environmental, spatial and temporal factors acting differently at the local, intermediate and regional spatial scales in Scarabaeinae beetle distribution in Neotropical region. It also highlights that the effect of these processes on species abundance in the Atlantic Forest also changes some aspects of the functional organization of dung beetle communities.

Functional diversity can be used as a complementary, but not substitute, approach to traditional measures of community responses for testing environmental and spatial effects on species distribution. The functional diversity approach may show different responses due to the ecological traits and functional diversity indices used, which will depend on the aim of the study. These new ways of gathering information on different species traits can be used to answer ecological questions about community assembly and ecosystem function [24], which is of great interest in the context of community ecology.

The community deconstruction approach allows us to identify sets of responses for different trait-based groups with distinct species composition. The deconstructive approach was useful to improve our understanding of dung beetle species responses to environmental, spatial and temporal effects. For each functional group category, we must take into account different assumptions to explain the responses, and it seems to be a fruitful way to test other hypotheses (beyond the importance of different processes) in shaping community structure [38]. Studies of metacommunities frequently mix “oranges with apples” [38], i.e., we generally expect that all species in a given community, which are composed of different sets of traits, respond the same way to different processes across different spatial scales, which is simply not true. The community deconstruction approach seems promising for a better understanding of how species respond to environmental and spatial effects in a metacommunity framework.

## Supporting Information

S1 Appendix Protocol for trait assignments. Dung beetle species were characterized in terms of four ecological attributes: food relocation behavior, diet, active period and biomass.
(DOCX)

S2 Appendix Protocol used to measure the environmental variables. Environmental variables were measured using the adapted point-centered quarter method.
(DOCX)

S1 Dataset Dataset of abundance and dry biomass of dung beetle species, environmental variables, and geographical coordinates. Samplings were performed in Brazilian Atlantic Forest, Santa Catarina, Brazil using baited pitfall traps from January to February 2012 and 2013.
(XLSX)

S1 Fig Rank abundance curve of dung beetle metacommunity (two years). Abundances are expressed as the percentage of the total abundance within the metacommunity. The dotted line indicates the inflection point of the curve used to classify the species into common or rare.
(EPS)

S2 Fig Relation between number of species and number of individual of each functional group of dung beetles. (EPS)

S3 Fig Permission letter. Permission request to publish Fig 1.
(TIF)

S1 Table Dung beetle traits. Identity and traits for 28 dung beetle species sampled in the Atlantic Forest from southern Brazil. NA: unavailable data. Other: unknown, but different from others.
(XLSX)

S2 Table Summary of environmental variables. Averages (mean or median, as appropriate), quartiles, minimum and maximum values. 25%: 25 percentile, 75%: 75 percentile; max: maximum recorded for the entire dataset.
(XLSX)

S3 Table List of Scarabaeine dung beetles species and total captures per years and area. ANH: Environmental Protection Area of Anhatomirim in Governador Celso Ramos (mainland); ITA: Permanent Protection Area of Itapema (mainland); PER: Lagoa do Peri Municipal Park, Florianópolis (island); RAT: Permanent Protection Area of Ratones, Florianópolis (island). T: total; GT: grand total.
(XLSX)
