# Supplementary material for: Scale-Dependence of Processes Structuring Dung Beetle Metacommunities Using Functional Diversity and Community Deconstruction Approaches
Source: PLoS One. 2015 Mar 30;10(3):e0123030. doi: 10.1371/journal.pone.0123030 (PMC4378897; doi:10.1371/journal.pone.0123030)
Supplement: S1 Appendix — Dung beetle species were characterized in terms of four ecological attributes: food relocation behavior, diet, active period and biomass. (DOCX) [file pone.0123030.s001.docx]

**S1 Appendix. Protocol for trait assignments.** Dung beetle species were characterized in terms of four ecological attributes: food relocation behavior, diet, active period and biomass.

Species were characterized in terms of ecological attributes: food relocation behavior, diet, diel activity and biomass. Protocols for trait assignments are described below. We also obtained additional information on dung beetle traits from the published literature and personal observations of specialists (Fernando Vaz-de-Mello, Universidade Federal de Mato Grosso, Brazil). Similar approaches were used by other authors [[1-3](#_ENREF_1)].

**Food relocation behavior:** Food relocation behavior was assigned following the classification of [[4](#_ENREF_4)] who categorized dung beetle species as rollers (telecoprids), tunnelers (paracoprids) or dwellers (endocoprids). Rollers build and roll food-balls over the soil until bury them. Tunnelers bury portions of food above or next the resource. Dwellers feed and nest inside or beneath the food source. Additional information was obtained from the published literature [[5-11](#_ENREF_5)]. When the information was unavailable, we opted to classify the species as the pattern shown by the genera according to several studies with the objective of minimizing the occurrence of NAs in our dataset. Species with different (unknown) patterns of those shown by the group were classified as “Other” (with different numbers at the end to differentiate them).

**Diet:** Species were categorized as coprophage or necrophage if at least 80% of the individuals were captured in traps baited with human feces or carrion, respectively. Species with similar numbers of individuals in both types of baited trap were considered generalists. Only dung beetle species with more than 10 individuals were assigned to diet categories based in our samplings. Additional information for species with few individuals sampled (< 10) was obtained from the published [[10](#_ENREF_10), [12](#_ENREF_12), [13](#_ENREF_13)] and unpublished literature [[14-16](#_ENREF_14)]. When the information was unavailable, we opted to classify the species as the pattern shown by the genera according to several studies with the objective of minimizing the occurrence of NAs in our dataset.

**Diel activity:** The dung beetle species were classified according to their period of fly activity in diurnal, nocturnal and diurnal-nocturnal following several authors [[2](#_ENREF_2), [3](#_ENREF_3), [17-23](#_ENREF_17)].

**Biomass:** All individuals of dung beetles were dried at 60°C for 72 h and weighed using a balance accurate to 0.0001 g to obtain the mean dry weight.

**References**

1. Barragan F, Moreno CE, Escobar F, Halffter G, Navarrete D. Negative impacts of human land use on dung beetle functional diversity. PLoS ONE. 2011;6: e17976.

2. Nichols E, Uriarte M, Bunker DE, Favila ME, Slade EM, Vulinec K, et al. Trait-dependent response of dung beetle populations to tropical forest conversion at local and regional scales. Ecology. 2013;94: 180-189.

3. Audino LD, Louzada J, Comita L. Dung beetles as indicators of tropical forest restoration success: Is it possible to recover species and functional diversity? Biol Conserv. 2014;169: 248-257.

4. Hanski I, Cambefort Y. Competition in dung beetles. In: Hanski I, Cambefort Y, editors. Dung beetle ecology. Princeton: Princeton University Press; 1991. pp. 305-329.

5. Halffter G, Matthews EG. The natural history of dung beetles of the subfamily Scarabaeinae (Coleoptera, Scarabaeidae). Folia Entomol Mex. 1966;12-14: 1-312.

6. Matthews EG. A taxonomic and zoogeographic survey of the Scarabaeinae of the Antilles (Coleoptera: Scarabaeidae). Mem Am Entomol Soc. 1966;21: 1-134.

7. Halffter G, Martínez A. Revisión monográfica de los Canthonina americanos (Coleoptera, Scarabaeidae) (2º. parte). Rev Soc Mex Hist Nat. 1967;28: 79-117.

8. Halffter G, Edmonds WD. Nesting behavior of dung beetles (Scarabaeinae). México, D. F.: Man and Biosphere Program - UNESCO; 1982.

9. Cambefort Y. Biogeography and evolution. In: Hanski I, Cambefort Y, editors. Dung beetle ecology. Princeton: Princeton University Press; 1991. pp. 51-67.

10. Cook J. A revision of the Neotropical genus *Bdelyrus* Harold (Coleoptera: Scarabaeidae). Can Entomol. 1998;30: 631-689.

11. Ratcliffe BC, Smith ABT. New species of *Canthonella* Chapin (Scarabaeidae: Scarabaeinae) from Amazonian Brazil. Coleopts Bull. 1999;53: 1-7.

12. Silva PGd, Vaz-de-Mello FZ, Di Mare RA. Attractiveness of different baits to Scarabaeinae (Coleoptera: Scarabaeidae) in forest fragments in the extreme south of Brazil. Zool Stud. 2012;51: 429-441.

13. Campos RC, Hernández MIM. Dung beetle assemblages (Coleoptera, Scarabaeinae) in Atlantic forest fragments in southern Brazil. Rev Bras Entomol. 2013;57: 47-54.

14. Marcon CB. Diversidade de besouros Scarabaeinae (Coleoptera: Scarabaeidae) em áreas de Floresta Ombrófila Densa em diferentes estágios sucessionais. Florianópolis: Universidade Federal de Santa Catarina; 2011. pp. 54.

15. Simões TO. Diversidade de besouros indicadores em fragmentos florestais nativos e exóticos na região de Anitápolis - Santa Catarina, Brasil. Florianópolis: Universidade Federal de Santa Catarina; 2013. pp. 63.

16. Bogoni JA. Associações entre besouros escarabeíneos e mamíferos de médio e grande porte da Mata Atlântica em Santa Catarina, Brasil. Florianópolis: Universidade Federal de Santa Catarina; 2014. pp. 97.

17. Hernández MIM. The night and day of dung beetles (Coleoptera, Scarabaeidae) in the Serra do Japi, Brazil: elytra colour related to daily activity. Rev Bras Entomol. 2002;46: 597-600.

18. Nichols E, Spector S, Louzada J, Larsen T, Amezquita S, Favila ME, et al. Ecological functions and ecosystem services provided by Scarabaeinae dung beetles. Biol Conserv. 2008;141: 1461-1474.

19. Edmonds WD, Zídek J. A taxonomic review of the Neotropical genus *Coprophanaeus* Olsoufieff, 1924 (Coleoptera: Scarabaeidae, Scarabaeinae). Insecta Mundi. 2010;0129: 1-111.

20. Campos RC, Anderson AB, Rovai AS, Tonetta D, Schmidt G, Martins IM, et al. Diversidade funcional em assembleias de escarabeíneos em um fragmento de Mata Atlântica. In: Doria JG, Gianuca A, Hernández MIM, editors. Ecologia de Campo no Parque Municipal da Lagoa do Peri. Florianópolis: Universidade Federal de Santa Catarina; 2011. pp. 25-31.

21. Lopes J, Korasaki V, Catelli LL, Marçal VVM, Nunes MPBP. A comparison of dung beetle assemblage structure (Coleoptera: Scarabaeidae: Scarabaeinae) between an Atlantic forest fragment and adjacent abandoned pasture in Paraná, Brazil. Zoologia. 2011;28: 72-79.

22. Schulze B, Hernández MIM. Diferenciação de nicho em besouros escarabeíneos de Mata Atlântica no Parque Municipal da Lagoa do Peri, Ilha de Santa Catarina. In: Fuentes EV, Hessel M, Hernández MIM, editors. Ecologia de Campo na Ilha de Santa Catarina. Florianópolis: Universidade Federal de Santa Catarina; 2012. pp. 255-262.

23. Medina AM, Lopes PP. Resource utilization and temporal segregation of Scarabaeinae (Coleoptera, Scarabaeidae) community in a Caatinga fragment. Neotrop Entomol. 2014;43: 127-133.
