# Supplementary material for: Scale-Dependence of Processes Structuring Dung Beetle Metacommunities Using Functional Diversity and Community Deconstruction Approaches
Source: PLoS One. 2015 Mar 30;10(3):e0123030. doi: 10.1371/journal.pone.0123030 (PMC4378897; doi:10.1371/journal.pone.0123030)
Supplement: S2 Appendix — Environmental variables were measured using the adapted point-centered quarter method. (DOCX) [file pone.0123030.s002.docx]

**S2 Appendix. Protocol used to measure the environmental variables.** Environmental variables were measured using the adapted point-centered quarter method.

The protocol used to measure the 20 environmental variables is described as follows. Measurement was performed using the adapted point-centered quarter method [[1](#_ENREF_1)] and was chosen for its simplicity and common use in phytosociological surveys [[2](#_ENREF_2)]. Variables measured: (1) circumference at breast height when diameter at breast height [DBH] > 5 cm), (2) height, (3) top diameter and (4) distance away from the nearest tree to the center of cross, (5-8) same measures for the greater tree distant up to 10 m, (9-12) similar measures for shrubs (circumference at ankle height when DBH < 5 cm and with a minimum height of 1 m), (13) land slope, (14) canopy cover, (15) percentage of leaf litter cover, (16) green cover and (17) exposed soil, (18) height and (19) dry biomass of leaf litter, and (20) altitude. The height of trees and shrubs was visually estimated with a ruler of 4 m length. Circumference and distance were measured with a millimeter tape measure. The percentage of litter, green cover, and exposed soil coverage in each quadrant was estimated in different classes (0-5, 6-25, 26-50, 51-75, 76-95, 96-100%) using a square of 1 m plastic pipes, placed about 20 cm away from the cross. Land slope was obtained at the center of the square using an inclinometer. Litter height was measured using a mm ruler at five points inside the square (near each corner and in the center). A five-inch square was constructed in the center of the 1 m square, and a portion of litter was removed. Litter was later dried in an oven (60°C for 72 hours) and weighed to obtain dry biomass. Using the same classes described above, the percentage of canopy cover was visually estimated using a hollow square of 10 cm side length, placed at a distance of 60 cm from the eye of the observer at a 20° angle in relation to the zenith [[2](#_ENREF_2)]. Altitude was obtained using a hand-held GPS at ground level. The basal area of trees and shrubs was calculated from the trunk circumference (based on the area of the circle). For each variable, a measure of central tendency was calculated based on the Shapiro-Wilk normality test. Thus, each environmental variable represented a central value (mean or median, as appropriate) of the four measures of each point; this was done to minimize the effects of visual estimation. A subset of the variables analyzed (three basal area, three heights, DBH) is used by the Conselho Nacional do Meio Ambiente (CONAMA), the Brazilian Council of Environmental issues, to characterize successional stages of Atlantic Forest in the state of Santa Catarina [[3](#_ENREF_3)].

**References**

1. Cottam G, Curtis JT. The use of distance measures in phytosociological sampling. Ecology. 1956;37: 451-460.

2. Ramos FA. Nymphalid butterfly communities in an amazonian forest fragment. J Res Lepid. 2000;35: 29-41.

3. Conselho Nacional do Meio Ambiente. Resolução CONAMA nº 4, de 4 de maio de 1994. Publicada no DOU nº 114, de 17 de junho de 1994, Seção 1, páginas 8877-8878. Brasília: Ministério do Meio Ambiente; 1994.
